# Introducing the “IJMPR Didactic Papers”

**DOI:** 10.1002/mpr.70000

**Published:** 2024-11-22

**Authors:** Hans‐Ulrich Wittchen, Daniel S. Pine, Freya Thiel

**Affiliations:** ^1^ Department of Psychiatry and Psychotherapy Ludwig‐Maximilians‐University Munich Germany; ^2^ National Institute of Mental Health, Intramural Research Program (NIMH‐IRP) Bethesda Maryland USA; ^3^ Institutsambulanz und Tagesklinik für Psychotherapie (IAP‐AVM GmbH) Dresden Germany

**Keywords:** epidemiology, psychiatry, psychology, statistical methods

1

Recent years have seen a range of statistical and methodological innovations of major relevance in mental health and psychopathology research that have become increasingly common in mental health research, with many theoretical and methodological developments quickly gaining traction. Given, however, that we receive many submissions that use these methods in a superficial and sometimes questionable way, *the International Journal of Methods in Psychiatric Research (IJMPR)* sees a need for didactic methods papers, prepared by distinguished expert panels, that illustrate these developments, critically review the theoretical background and empirical practice and provide guidance for their use in the future.

In response to this need IJMPR has decided to launch a new type of article called “*IJMPR Didactic Papers*.” We have identified various critical topics and have commissioned the preparation of such didactic articles that will be published after the mandatory peer review together with regular accepted paper submissions in selected issues of IJMPR.

In this issue, we present the first of this new series of didactic papers on the topic of “*Network Analysis: An Overview for Mental Health Research*” (*Briganti et al.* 2024).

Written by a large panel of outstanding international experts, guided by Giovanni Briganti, this article illustrates contemporary practices in applying network analytical tools, bridging the gap between network concepts and their empirical applications. The authors explain how to use graphs to construct networks representing complex associations among observable psychological variables, they discuss key network models, including dynamic networks, time‐varying networks, network models derived from panel data, network intervention analysis, latent networks, and moderated models as well as Bayesian networks and their role in causal inference with a focus on cross‐sectional data. They value of this outstanding exposition is further enhanced by a discussion of how network models and psychopathology theories can meaningfully inform each other and a conclusion that summarizes the insights each technique can provide in mental health research.

In subsequent issues over the next 2 years, IJMPR will address in a similar way other critical topics, such as on “Mendelian Randomization,” “Machine Learning” and “Causal Forests,” each prepared by distinguished expert groups.

The special characteristic of all “IJMPR‐Didactic papers” are that they can be longer than usual submissions in order to allow for practical guidance, and to highlight the “Do's and Don't's,” with the ultimate goals of making readers familiar with such innovative methods and strategies and promoting the appropriate use of such methods in future research. Assuming that the *IJMPR Didactic Papers* hopefully will become a key reference standard for a wider audience in the future, we also plan with our publisher Wiley to create a “Special Collection of IJMPR Didactic Papers,” available online on the Wiley journal webpage, once the first three are published.

Although Didactic Papers are currently typically prepared on invitation only by the IJMPR Editorial board, we certainly welcome also proposals for such methodological topic papers by our readership and colleagues.

We hope that our initiative of “IJMPR Didactic Papers” will be successful by reaching a wider audience and enhancing the quality of future research in psychiatry and the mental health field.

## CONFLICT OF INTEREST STATEMENT

The authors have no conflict of interest to declare.

## Data Availability

Not applicable.
